# The noncoding RNA revolution—three decades and still going strong!

**DOI:** 10.1002/1878-0261.12418

**Published:** 2019-01-07

**Authors:** George A. Calin

**Affiliations:** ^1^ Experimental Therapeutics and Leukemia Departments The Center for RNA Interference and Non‐Coding RNAs University of Texas, MD Anderson Cancer Houston TX USA

Revolutions bring great changes to societies. The noncoding RNA revolution began with the seminal discovery of *Caenorhabditis elegans* small noncoding RNAs, known as microRNAs, by Ambros, Ruvkun, and colleagues in 1993; this revolution has wrought huge changes in all fields of biology and medicine by identifying a new layer of gene regulation. The most unbiased proof of their impact is the publication of over 100 000 papers on the topic of short or long noncoding RNAs, reporting their roles in cell fate, tissue organization, organism homeostasis, and circadian rhythms, their use as biomarkers, their involvement in a plethora of disease mechanisms, and, starting recently, their potential as drugs or drug targets in several clinical trials for various disorders.

Due to this unprecedented coverage of the role of noncoding RNAs in almost all aspects of biology and pathology, this special issue of *Molecular Oncology* on the topic of noncoding RNAs is focused on specific issues that have practical significance, rather than trying to encompass the full biology of these molecules.

## In this issue

Joanne Weidhaas and Ye Yuan review microRNA‐associated polymorphisms that have been shown to be important biomarkers of cancer risk, prognosis, and treatment outcomes. This has great translational significance, as these predictive biomarkers can be used for patient selection into treatment groups in clinical trials (Yuan and Weidhaas, 2018).

Anke van den Berg, Joost Kluiver, and colleagues cover the important topic of crosstalk between MYC and noncoding RNAs, and the significance for potential new therapeutic approaches in human cancers (Swier *et al*., 2018).

The review by Spina and Rossi gives an overview of the mutation landscape in the most frequent type of human leukemia, chronic lymphocytic leukemia, with a particular focus on noncoding lesions of the genome (Spina and Rossi, 2018).

Martin Pichler and colleagues review the role of a highly expressed ncRNA, NEAT1, in carcinogenesis and discuss some contradictory reports related to its potential interaction with microRNAs (Klec *et al*., 2018).

Cristina Lo Nigro and colleagues discuss the roles of lncRNAs as regulators of cancer immunity, a particularly important topic in light of the recent success of several types of immunotherapies (Denaro *et al*., 2018).

Manuela Ferracin and colleagues report on the involvement of certain small and long noncoding RNAs in cutaneous melanoma, and the diagnostic and prognostic potential of microRNAs for this disease (Riefolo *et al*., 2018).

Ioana Berindan and colleagues focus on the roles of the miR‐181 family, one of the most frequently deregulated families of microRNAs in lung cancers, and their potential use as biomarkers and therapeutic targets for this deadly malignancy (Braicu *et al*., 2018).

In closing, we hope that this special issue will not only answer many of our readers’ questions on this topic, but also raise new ones that will open new venues of research and lead to significant discoveries in this ongoing revolution.
